# Recent Advances in the Aggregation Behavior of Nanoplastics in Aquatic Systems

**DOI:** 10.3390/ijms241813995

**Published:** 2023-09-12

**Authors:** Antonio Cid-Samamed, M. S. Diniz

**Affiliations:** 1 Physical Chemistry Department, Faculty of Sciences, University of Vigo, 32004 Ourense, Spain; 2 i4HB–Associate Laboratory Institute for Health and Bioeconomy, NOVA School of Science and Technology, NOVA University Lisbon, 2819-516 Caparica, Portugal; mesd@fct.unl.pt; 3 UCIBIO–Applied Molecular Biosciences Unit, NOVA School of Science and Technology, NOVA University of Lisbon, 2829-516 Caparica, Portugal

**Keywords:** nanoplastics pollution, aggregation, surfactant-based nanoplastic remediation, surface chemistry, removal efficiency

## Abstract

This short review aims to critically discuss the recent advances in supramolecular chemistry to achieve the aggregation of nanoplastics in aquatic systems. Polymer modification provides a vital tool for designing novel and ad hoc synthesized surfactants with properties tuned for some specific applications (e.g., stimuli-responsive nanomaterial, conducting polymers), mainly to aggregate other polymers from the environment. Far from the typical use of surfactants, which ease the dispersion of insoluble molecules in water media or aid solubilization of insoluble molecules on local media, in this case, nanoarchitectonics serve researchers to design surfactants with a focus on the capture of nanoplastics from the environment. Additionally, monovalent and divalent salt additions aided NPs in coagulating in the aquatic systems. Finally, the latest research on NPs’ removal efficiency on wastewater treatment plant is reviewed to summarize the advances.

## 1. Introduction

Since 1950, plastics production has increased from 1.5 million tons in 1950 to 370 million tons in 2020 [1,2]. They can be found in the most remote places on the planet as microplastics (MPs). The term “microplastic” refers to plastic particles smaller than 5 mm in size. Their origin attends two different classifications: primary and secondary microplastics. Primary microplastics are directly synthesized and are generally included in hygiene or cosmetic products [3,4]. Secondary microplastics arise from the fragmentation of larger plastics by biodegradation or physicochemical processes, such as oxidation, hydrolysis, temperature, or photodegradation.

Microplastics can be further degraded into nanoplastics (1–100 nm). Therefore, this review focuses on microplastics and these smaller nanoplastics and their colloidal behavior in water.

Microplastics and nanoplastics can be found in different locations and compartments. They are found in arctic ice, soil, land niches, or even foods. The atmosphere is also not free of microplastics (MPs), as documented studies show a daily deposition of 366 particles per square meter in the Pyrenees region of southern France, and there were no obvious sources of microplastics within a 100 km radius [5]. Seas and oceans receive up to 12 million tons of plastic debris annually, representing up to 90% of the total waste collected in these ecosystems [6]. The best estimates show that 80% of ocean plastics come from land-based sources, while the remaining 20% comes from marine sources, such as the fishing industry, nautical activities, and aquaculture [7].

**Figure 1:**
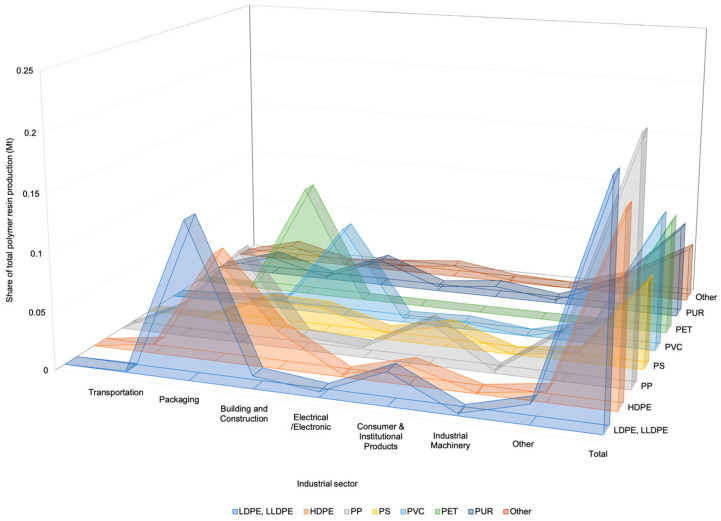
Share of total polymer resin production according to polymer type and industrial use sector calculated from data for Europe, the United States, China, and India covering the period 2002–2014 (from data of [9]).

Microplastics were a matter of concern for the first time in the 1970s when found in the Northwest Atlantic [8], but little attention was paid to them until they became an inherent problem. Figure 1 shows the share of total polymer resin production according to polymer type and industrial use sector calculated from data for Europe, the United States, China, and India covering the period 2002–2014 [9].

The distribution of plastic accumulation can be classified into three environments: the shoreline, coastal areas, and offshore. Shorelines store two-thirds of floating macro-litter released in marine systems from 1950 to 2015, gathering 40 million tons of microplastic. Coastal waters up to 200 m deep accumulated 80 million tons of microplastics in the same period. The offshore (>200 m deep) was the most contaminated region, accumulating one million tons of macroplastics and 0.5 million microplastics. Far from expected, most microplastic deposition comes from the 1990s and earlier. Therefore, the shoreline is the marine system most affected by microplastic pollution since most macroplastic waste goes offshore [10].

**Figure 2:**
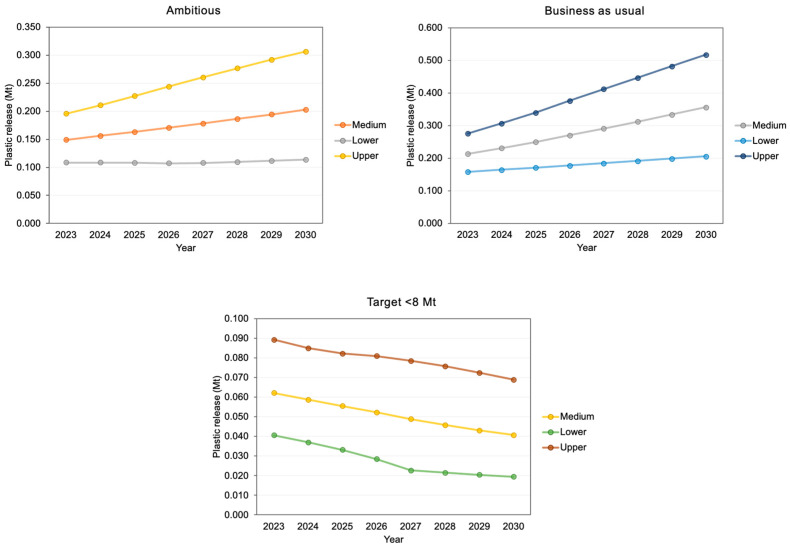
Average plastic release into aquatic ecosystems (including lakes, rivers, and oceans) for each scenario medium, upper, and lower values of the countries studied in 2023, and prediction until 2030 of the same taking into account an ambitious project, business as usual, and with the objective <8 Mt. (Data extracted from the S4 file of ref. [11].)

Figure 2 shows the average plastic release into aquatic ecosystems (including lakes, rivers, and oceans) for each scenario medium, upper, and lower values of the countries studied in 2023 and prediction until 2030 of the same, taking into account an ambitious project, business as usual, and with the objective <8 Mt [11].

Plastics at these depths will take much longer to fragment due to the absence of ultraviolet (UV) penetration and much colder water temperatures [6]. Likewise, MPs are distributed in three aquatic environments (sediment, water column, and water surface) in size. Larger-sized MPs usually remain in surface layers, while small MPs (<100 μm) accumulate in deeper water layers [12].

The density of NPs varies depending on their composition. The composition of plastics is highly variable, depending on their intended use. It has been previously reported that the six most widely used types of polymers globally are polypropylene (PP), polyethylene (PE), polyvinyl chloride (PVC), polyethylene terephthalate (PET), polystyrene (PS), and polyurethane (PU) [13].

Recent scientific advances have shown a new problem associated with plastics and NPs since they can work as vectors for non-polymeric environmental pollutants. 

Concerns about their potential impact have been classified into two categories. The first concern is the impact of the release of persistent bio-accumulative and toxic compounds (PBTs). This group includes molecules such as polyaromatic hydrocarbons (PAHs), polychlorinated biphenyls (PCBs), hexachlorocyclohexane (HCH), the insecticide DDT, and other persistent organic pollutants (POPs). These toxicants act as endocrine disruptors, and their metabolites might cause genetic mutations, resulting in cancer. Bioaccumulation in organisms as they enter the food chain magnifies exposure levels and food webs, whose effect differs for each compound and is usually unknown. The second concern involves additives in the plastics manufacturing processes and the release of additives related to the manufacture and breakdown of plastic, monomers (such as BPA), and additives [14,15]. These additives include phthalates, styrene monomers, and bisphenol A (BPA) [7]; BPA is one of the most synthesized chemicals in the world. As a potential endocrine disruptor, it binds to nuclear estrogen receptors, mimicking endogenous hormones’ effects. The effects of an organism’s exposure to this compound may range from cancer development to reduced fertility. MNPs’ harmful potential involves microbial transfer along distant geographical regions, as microorganisms form biofilms on plastic surfaces. The influence of biofilms is not yet fully understood, despite evidence that biofilms can modify plastic properties, changing the fate of the molecules [16]. In addition, previous research has highlighted the potential impact of these additives in plastics on humans, with the most likely routes of entry being ingestion (through contaminated food or trophic transfer), inhalation, or skin contact [17].

**Figure 3:**
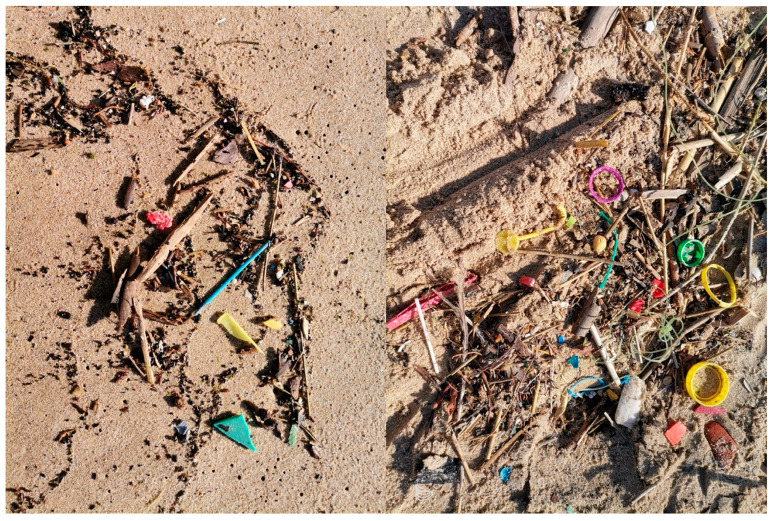
Debris on a Portuguese beach (Lisbon region) shows different plastic sizes and shapes.

As an example of plastic pollution, we present in Figure 3 representative images of a Portuguese beach:

This review aims to:

- Summarize the recent research on MNPs pollution in aquatic environments.
- Discuss and clarify the supramolecular approaches and methods to promote the removal of NPs from aquatic systems.
- Summarize and discuss the recent approaches and methods applied in WTPs to minimize the release of NPs into the water cycle.
- Summarize the latest advances and future perspectives on NPs removal from aquatic systems.

## 2. Methodology

### 2.1. Aggregation Behavior of Nanoplastics: The Effect of Surface Chemistry and Physical Chemistry of the Surrounding Media

In this section, a summary of different approaches and methods are reviewed, focusing on the aggregation behavior of nanoplastics considering the impact of several factors such as surface chemistry of nanoplastics, physico-chemical conditions of surrounding media, and proposed supramolecular solutions to remove NPs from the aquatic systems.

The functionalization of nanoparticles can tune the nanoparticle suspensions’ stability. In general, even the most advanced nanomaterials lose their function if the suspensions aggregate and settle. Still, functionalized nanomaterials remain monodisperse and maintain their function with the coating agents designed for specific solution chemistries. Advanced functionalization methods require sufficient control over suspension stability with minor changes to a single, controllable variable. Some examples of these materials include stimulus-responsive polymers for functionalization, which are based on a bulk solution physicochemical threshold (temperature or pH) that changes from a stable (monodisperse) to an aggregated state. The authors also highlighted the additional factors that rule nanoparticle stability, such as the core composition, surface chemistry, and solution conditions [18]. The nature of nanoplastics depends on the polymer used to synthesize the engineered nanomaterial. The functional groups used to label the nanoplastics also affect their colloidal behavior. 

On the other hand, surrounding media pH and ionic strength, divalent cations, and dissolved organic carbon influence the aggregation behavior and toxicity of nanoplastics. For instance, Zhang et al. reported on the aquatic behavior of polystyrene NPs with different functional groups [19]. The authors systematically studied the roles of water chemistry (dissolved organic carbon (DOC), pH, and divalent cations) and particle surface chemistry that rules the aqueous stability; thus, aggregation and toxicity of engineered nanoplastic particles in reproduced natural environmental conditions. These authors used polystyrene latex NPs (PLNPs) with three different functional groups, viz. original (uPLNPs), amine-modified (aPLNPs), and carboxyl-modified (cPLNPs), as a model to investigate its behavior. The results indicate that only the DOC’s presence augmented the surficial charge and displayed trivial results on the PLNPs size distribution in aqueous suspensions. The divalent cations (Ca^2+^ and Mg^2+^) were observed to decline the surficial charge and raise the size of the PLNPs. The coexistence of DOC and the divalent cations enhanced the aggregation degree of the PLNPs in the water column. The surficial alteration and pH were sensitive factors affecting the PLNPs’ stability through long-term suspension when DOC and the divalent cations co-occurred. Direct visualization further confirmed the conclusions about the joint effects of solution and surficial chemistry parameters. Additionally, in situ transmission electron microscope (TEM) imaging showed that the augmentation of PLNP aggregation in the presence of DOC and the divalent cation was caused by bridge formation. The co-occurrence of DOC and the divalent cations produced noteworthy aggregation and sedimentation of PLNPs. Moreover, the boost of PLNP aggregation when DOC was present was related to forming bridges in the divalent cation solutions. 

The influence of the counterion nature on the stability of hydrophobic latex particles has also been reported [20]. The authors reported the influence of monovalent counterions’ lyotropic nature in achieving the inversion of the stability sequence of negatively charged (from sulfate free radicals (SO_4_^−^)) hydrophobic latex particles. Based on aggregation kinetics experiments, the influence of anions, acting as co-ions, was notably evidenced. The studied monovalent co-ions were IO^−^, F^−^, Cl^−^, NO^−^, I^−^, and SCN^−^. The ordering of the ccc (critical coagulation concentration) values for these co-ions was reversed when using the kosmotrope sodium and the chaotrope potassium or ammonium counterions. Their results suggested the competitive behavior of co-ions and counterions within the inner layer at the hydrophobic particle–aqueous solution interface.

Previous research reported that polyethylene (PE) particles with radii sizes between 200 and 800 nm could be synthesized with or without a surfactant using emulsions of toluene in water with PE dissolved in toluene. The authors reported that the particle size did not depend much on the amount and type of surfactant (Tween 60, Tween 80, and a biosurfactant). It must be noted that PE particles remained stable in an aqueous solution for at least three months and did not aggregate in water containing sea salt at the average marine concentration [21].

Cai et al. reported on the effects of natural organic matter (NOM) and inorganic ions on the aggregation of polystyrene (PS) NPs. The aggregation of NPs is a crucial issue due to the nature of NPs in the environment. PS NPs remained stable on a wide range of ionic strength solutions of NaCl and CaCl_2_ and low ionic strength FeCl_3_ solution. Summarizing their results, comparing the insignificant effects of CaCl_2_ and NaCl over a wide variety of ionic strengths on PS NPs aggregation, Fe (III) with far poorer ionic strength in water could promote a fast and apparent aggregation of PS NPs. Additionally, NOM’s effect on the aggregation of PS NPs was negligible for both salt solutions, though NOM could hinder or promote the aggregation of PS NPs in FeCl_3_ solutions at different ionic strengths. The authors concluded that the PS NPs’ aggregation with Fe (III) leads to that with Na(I) and Ca(II), and the subsequent effect of NOM depends on particular solution conditions [22].

The surface functionalization is a crucial parameter determining the fate of 50 nm NP in the reported conditions of the experimental design proposed by Tallec et al. [23] and possibly in aquatic environments. The data reported in this research, complemented with previous research [22] on plain PS, underlines the significance of the characterization of NPs by studying at least size and coating in relevant environments to improve the experiments. It is also required to understand the effect of other environmental conditions on NP behavior, particularly weathering particles, affecting their previously observed properties with MPs [14].

The aggregation kinetics of different surface-modified polystyrene NPs in mono- and di-valent electrolytes were studied to clarify their behavior in the aquatic environment [24]. Three monodisperse NPs, including unmodified NPs, carboxylated NPs, and amino-modified NPs, among one polydisperse NPs produced by laser ablation of polystyrene films, were used as models to clarify the significance of morphology and surficial groups. The results displayed that aggregation kinetics of negatively charged PS-Bare and PS-COOH followed the DLVO theory in CaCl_2_ and NaCl solutions. The addition of Suwannee River natural organic matter (SRNOM) blocked the aggregation of PS-Bare and PS-COOH in NaCl by steric limitation. Conversely, in divalent electrolytes, their stability was increased at low concentrations of SRNOM (less 5.0 mg C L^−1^), while it worsened at high concentrations of SRNOM (over 5.0 mg C L^−1^) because of the interparticle bridging effect caused by Ca^2+^ and carboxyl groups of SRNOM. The cation bridging effect was also observed for PS-laser in high concentrations of divalent electrolytes and SRNOM. The adsorption of SRNOM could counteract or even inverse surface charges of positively charged PS-NH_2_ at high concentrations, thus enhancing or inhibiting the aggregation of PS-NH_2_. The steric repulsion imparted by the surface modification showed no synergetic weight of Ca^2+^ and SRNOM on the aggregation of PS-NH_2_. Their results underline that surficial charge and its alteration notably impact the aggregation behaviors of NPs in aqueous media.

Characterizing the environmental conditions is paramount to understanding the behavior of NPs in aqueous media. This need was reported by Wu et al. [25] for four polystyrenes NPs with different functional groups, and charges (PS, PS-COOH, n-PS–NH_2_, p-PS–NH_2_) were selected to investigate the effect of humic acid (HA) and salinity on their aggregation behavior and toxicity. The authors concluded that the salinity enhanced the NPs aggregation, whereas HA primarily acted as a stabilizer on the three negatively charged NPs but played a different role in the aggregation process of PS-NH_2_. The combined effect mainly depended on their concentration ratio. The Derjaguin–Landau–Verwey–Overbeek (DLVO) theory explains the aggregation phenomena.

During the travel of NPs from freshwater systems to estuarine and oceanic regions, they can interact with dissolved organic matter (DOM) to form microgels. Microgel formation is ubiquitous in aquatic systems, serving as a shunt between DOM and particulate organic matter (POM) and playing crucial roles in particle aggregation, sedimentation, and pollutant transport. Previous research reported that 25 nm polystyrene NPs in the lake and river water could promote POM (microgel) formation and hasten the DOM–POM transition [26]. The authors also adjusted different water samples’ salinities to mimic circumstances based on plastic transportation in waters flowing from rivers to seas. The results indicate that polystyrene nanoparticles can interact with organic matter to form large organic particles, which may undergo further settling in response to specific salinity levels. Polystyrene-induced microgel formation appears to involve the hydrophobic interactions between plastics and DOM. They showed that NPs alter the DOM–POM shunt to cause unexpected perturbations in aquatic ecosystems’ functionality.

Conversely, recent research reported [27] on the effects of pH, various metal cations (K^+^, Na^+^, Ba^2+^, Ca^2+^, Mg^2+^, Fe^3+^, Al^3+^), aging, and extracellular polymeric substances (EPS) on the aggregation of polystyrene nanoplastics (nano-PS) in aqueous solutions were methodically assessed based on aggregation kinetics experiments and Derjaguine–Landau–Verweye–Overbeek (DLVO) theoretical calculation. Metal cations’ concentration, valence, and hydration ability jointly affected nano-PS aggregation. The critical coagulation concentration (CCC) of nano-PS was appreciably higher than the ionic strengths in aquatic environments, denoting that the aggregation rate of nano-PS is comparatively low. The aggregation kinetics experiments’ results were consistent with DLVO theory, which showed that nano-PS’s energy barrier depended on electrostatic repulsion and Van der Waals forces and was augmented with pH. Nano-PS was unnaturally aged by UV-H_2_O_2_, which reduced the hydrophobic nature of the particle surfaces, consequently increasing the stability of the NPs. EPS (excreted from *Chlorella Pyrenoidosa*) decreased nano-PS aggregation rates due to steric effects, confirmed by the extended DLVO model. Their results underline the high stability of nano-PS in aquatic environments. The aggregation kinetics of nano-PS clearly showed reaction- and diffusion-limited regimes in the presence of different metal cations. The CCC for different valence ions was proportional to z^−6^ to z^−3^, consistent with the Schulze–Hardy rule. The decrease in the energy barrier and second energy minimum caused by metal cations contributed to nano-PS aggregation. The CCC of the nano-PS in the presence of NaCl at different pHs agreed with the DLVO theory. The aging-induced C=O groups’ formation on the nano-PS surface decreased the aggregation degrees. The interaction among the nano-PS particles in the presence of EPS was dominated by steric hindrance, electrostatic repulsion forces, and Van der Waals forces. These results help understand the aggregation behavior of nano-plastics in aquatic environments.

Given previously described research, the aggregation of NPs depends on the polymer nature of the NPs, surface chemistry (with or without functional groups), salinity and surrounding media conditions (pH, ionic strength), presence of organic matter, both polymers (acting as stabilizers or capping agents), or natural occurring matter (including, dissolved organic matter, dissolved organic carbon, and extracellular polymeric substances).

### 2.2. Proposed Supramolecular Solutions to Remove NPs from the Aquatic Systems

Some solutions have been proposed to modify latex NPs by the layer-by-layer technique for drug loading and release [28]. The proposed strategy is advantageous compared with other synthetic routes because, at all steps, only water is used as a solvent, thus avoiding organic solvents. Poly(vinyl acetate) (PVAc) latex particles were synthesized by mini-emulsion polymerization of vinyl acetate using sodium dodecyl sulfate (SDS) as an emulsifier. This latter provided a negatively charged surface to latex NPs and was favorably employed as a core to be covered with natural polyelectrolytes (alginate and chitosan) through layer-by-layer deposition. A simple diffusion process/electrostatic interaction prompted the amoxicillin (an antibiotic) trap within the multilayer shell was prompted by a simple diffusion process/electrostatic interaction. The loaded nanocarriers covered by (chitosan/alginate) n-chitosan layers showed a high zeta-potential value (about +35 mV). Dynamic light scattering (DLS) and transmission electron microscopy (TEM) highlighted a spherical shape and a size under 200 nm. Lastly, the authors evidenced the pH-triggered drug release (tocopherol acetate and amoxicillin) and the influence of the parameter forming the multilayer, i.e., number and cross-linking. The results demonstrate that the elaborated latex-core polysaccharide-coated nanoparticles are rising as multifunctional nanocarriers.

Cao et al. [29] reported using an ionic surfactant, sodium di-2-ethyl hexyl sulfosuccinate [30,31,32] (AOT), to activate or charge PS nanoparticles of different sizes: 141, 311, and 535 nm. The behavior of nanoplastics showed that the PS/AOT/water system’s charging mechanism was adsorption-dominated, revealing the importance of the association between the activation of the particle surfaces and particle charging.

Mitrano et al. [33] reported a method to synthesize NPs doped with a chemically trapped metal used as a tracer, which offers a robust route to detect and quantify nanoplastics more comfortably and accurately in complex media. This approach is beneficial when it is intended to study the simulation of the activated sludge process of a municipal wastewater treatment plant (WWTP) to deepen the understanding of the fate of NPs. Most of the particles were bound to the sludge, reaching an average recovery of over 93% of the spiked material. Authors expect their approach to be further developed to assess the fate, transport, mechanistic behavior, and biological effects in various systems at different scales. Another recent study reported nanoplastics (PSNPs) interactions with four typical minerals, comprising goethite, magnetite, kaolite, and montmorillonite, studied in the aqueous phase. Destabilization of PSNP colloidal suspensions occurred by the presence of the positively charged magnetite or goethite, while the suspension remained stable or not affected by the negatively charged kaolinite and montmorillonite. This latter signifies the strong electrostatic interaction between PSNP and both iron oxides. Additionally, another three metal ions (MnO_2_, Al_2_O_3_, and SiO_2_) confirmed the significance of electrostatic interactions on the stability of PSNP suspension [34]. The efficient adsorption of PSNP on magnetite or goethite due to the strong electrostatic interaction was confirmed by TEM analysis and batch adsorption experiments. Moreover, strong hydrogen bonding between the hydroxyl group (γ-FeOOH) of goethite and PSNP, contributing to the higher adsorption of PSNP on goethite than magnetite, was confirmed by FTIR and two-dimensional correlation spectroscopy. These results clarify the interactions of PSNP with mineral surfaces and the potential outcome of PSNP on natural conditions in aquatic systems.

The latest research on aggregation and stability of surface-modified nano polystyrene in natural and synthetic waters has been recently reported by Wang et al. [35]. The authors confirmed the effect of pH, inorganic ions, and NOM presence on the ζ-potentials, *D_H_*, and aggregation behavior of PSNPs. The critical coagulation concentrations of PSNPs were determined to be 158.7 mM NaCl, 12.2 mM CaCl_2_, and 80.0 mM Na_2_SO_4_. Due to a stronger charge screening effect, Ca^2+^ became more influential than Na^+^ in destabilizing PSNPs. NOM hinders or promotes the aggregation of PSNPs as a function of the presence of ions for monovalent and divalent ions. NOM was the most significant factor affecting the NPs aggregation—for high ionic strength and NOM content water—probed through initial and long-term stability studies. PSNPs would remain highly suspended in all freshwaters, even in wastewater, while aggregating quickly and depositing in seawater. The results confirmed that steric repulsions and electrostatic interactions dominated the aggregation of PSNPs. At low ionic strength, co-ions played a crucial role in providing charges on nanoplastics surfaces, while at high ionic strength dominated the electronic double-layer compression and charge neutralization by counterions.

#### Characterization Methods

Concerning methodology, different techniques must be underlined, such as microscopic and spectroscopic methods, mass spectrometry-based methods, and other techniques. For instance, Zhang et al. [19] reported on the aquatic behavior of polystyrene NPs with different functional groups functionalization and, by using DLS, TEM, and UV-Vis spectroscopy, confirmed the effect of amino and carboxyl-modified PLNPs under different pH conditions. The authors found that the hydrodynamic diameter of PLNPs decreased in the order uPLNPs > aPLNPs > cPLNPs. Additionally, the enhancement of PLNP aggregation when DOC was present was associated with the formation of a bridge in the divalent cation solutions. Using the same methods, Trompette et al. [20] studied the aggregation kinetics by measuring the change in the absorbance at 600 nm as a function of time to determine the coagulation critical concentration and, therefore, the stability ratio of the colloidal system. Furthermore, the authors found the competitive behavior of the implied co-ions and counterions within the inner layer at the hydrophobic particle−aqueous solution interface.

Lagarde et al. [21] adopted an opposite strategy; in their research, they synthesized PE nano and microplastics to be used as models to assess the toxicological impact of micro- and nanoplastics on marine biota. After the emulsion-based synthesis of PE particles, they determined the size and shape by CLSM and the weight loss during degradation using TGA, varying the temperature from 40° to 800 °C at a rate of 20 °C/min. The crystalline fraction of the particles was measured by DSC. Lastly, the bioavailability of PE particles in the presence of *Daphnia magna* was followed for 72 h to test the intake, and it was visualized using microscopy under polarized light. Cai et al. [22] studied the effects of inorganic ions and NOM on the aggregation of NPs, using NaCl, CaCl_2_, and FeCl_3_ salts recording the ζpot by DLS and the aggregation by time-resolved DLS; to clarify the effect of NOM on PSNPs aggregation, they used fluorescence microscope images 10 min after blending PSNPs and NOM. The researchers discovered that FeCl_3_ was the only salt that affected the NPs’ aggregation. Moreover, the impact of NOM on the NPs’ aggregation behavior depended on specific dissolution conditions. Tallec et al. [23] studied the aggregation behavior of three different surface functionalization of PSNPs (plain, amino, and carboxyl) recording by DLS Z-average (nm), aggregation state (PDI (A.U.), and the mean surface charge (ζpotential(mV)). Moreover, they assessed the effects of environmental conditions, following the impact of the media (pH), salinity, and organic matter (DOM- humic acid). They concluded that surface functionalization is a major parameter in the experimental design and aggregation behavior of NPs.

The effect of monovalent and divalent electrolytes on PSNPs aggregation was reported by Liu et al. [24] using laser ablation of polystyrene films to synthesize PSNPs. They also functionalized commercial PSNPs for a total of three different PSNPs (PS-Bare, PS-COOH, and PS-NH_2_) to study. The ζpot and *D_H_* were recorded by DLS, and the dissolved organic carbon (DOC) was measured by the total organic carbon analyzer. Time-resolved DLS was used for batch experiments (using a wide range of CaCl_2_, NaCl, and SRNOM) and was a suitable tool to accurately detect CCC values and study the aggregation behavior. 

Wu et al. [25] used four nanosized PS NPs (PS, PS-COOH, n-PS-NH_2_, and p-PS-NH_2_) to study the aggregation behavior. They recorded the TOC in the presence of the humic acid elemental composition of the dispersion of PSNPs, HA, and NaCl. They then measured the *D_H_* and ζpot by DLS. A particular mixture was prepared for exposure tests with *Daphnia magna* by mixing the previous suspensions with ethanol and then measured by DLS using PALS and multi-frequency measurement. Fluorescence quenching in the presence of HA was performed by fluorospectro-photometer. The same procedures were followed to detect the effect of salinity on the aggregation behavior of NPs singly and combined with HA.

R.-F. Shiu et al. [26] collected water samples from the Lake of Yosemite and Hartley Slough River (California) and then analyzed pH, calcium and magnesium content, and salinity. Additionally, DOC concentration was determined as total carbon by catalytic high-temperature oxidation using a TOC analyzer. Then, microgel size measurement was followed by DLS and flow cytometry, with the latter using DI water as blank and fluorescent microbeads as control.

Y. Mao et al. [27] reported the high stability of PSNPs in aquatic environments. A dispersion of commercial PSNPs (diameter 100 nm) was prepared in ultrapure water electrostatically stabilized, displaying a ζpot of −30 mV and PDI of 0.2. Size distribution was recorded by DLS, morphology was determined by SEM, and components of NPs were followed by ATR-FTIR. Then, the aging process was performed in an aging chamber with two UV lamps. Next, the PS dispersion and H_2_O_2_ were added to a quartz vial, and XPS, ATR-FTIR, and TGA were used to assess the changes in the physicochemical properties of the PSNPs. Moreover, the organic components and functional groups of EPS (*Chlorella pyrenoidosa*) were measured by a three-dimensional fluorescence excitation–emission matrix (3D-EEM) and ATR-FTIR. The time-resolved hydrodynamic diameter was recorded by time-resolved DLS; from the autocorrelation function by cumulants method, authors obtained the intensity-weighted particle size distributions as z-average hydrodynamic size. Additionally, to assess the aggregation kinetics, the suitable electrolytes and EPS solutions were added to PSNPs suspensions to a final volume of 2 mL for each DLS measurement. To calculate the interaction energy between colloids and explain the aggregation mechanism, DLVO theory and extended DLVO were used.

Previous research from S. Belbekhouche et al. [28] started by synthesizing negatively charged surface poly(vinyl acetate) latex NPs using mini-emulsion polymerization with SDS as an emulsifier, then authors used LbL (layer-by-layer) deposition to obtain core/shell NPs; first, the NPs were covered with polycation, chitosan, and, after the polyanion, alginate. The dispersion of multilayered PSNPs was then summited to dialyses, adding glutaraldehyde to cross-link the polyelectrolyte layer. Authors added different drugs into modified NPs; i.e., PvAcNPs were loaded with tocopherol acetate, and then the efficiency of bilayer-based NPs on entrapment of amoxicillin and tocopherol acetate was assessed. The PSNPs dispersions were characterized by DLS, to record the size and ζpot of the particles. Molecular weight distributions and average molecular weights were measured by size-exclusion chromatography (SEC) coupled on-line with multi-angle light scattering (MALS) and differential refractive index (DRI) detectors. Additionally, absorbance spectra were followed by a UV-vis spectrophotometer. Micrographs of particles were obtained with TEM-equipped XEDS for chemical analyses.

Cao et al. [29] assessed the charge-mediating of PSNPs using AOT surfactant in aqueous solutions [30,31,32]. PSNPs were obtained with three different sizes: 141, 311, and 535 nm. Dispersity and morphology of the particles were recorded by SEM micrographs. The conductivity and pH of AOT/water and PS/AOT/water systems were recorded to measure the conductivity as a function of the concentration of AOT. Moreover, to determine the electrophoretic mobility, a PALS was used. 

Mitrano et al. [33] reported the use of metal-doped NPs to study the behavior in complex environmental systems. The authors followed different characterization methods, such as imaging (SEM, STEM-EDX), to determine the size, morphology, structural and elemental chemical mapping, and sizing (DLS, electron microscopy), as well as to record metal (Pd) incorporation (ICP-MS).

Y. Zhang et al. [34] studied the interactions between PSNPs and montmorillonite, kaolinite, magnetite, and goethite in aqueous systems. The major role of electrostatic interaction in the stability of PSNP suspension was confirmed by incubation with other metal oxides (MnO_2_, Al_2_O_3_, and SiO_2_). The samples were characterized by TEM, SEM, and batch adsorption experiments, which showed the PSNPs effectively adsorbed on goethite and magnetite to the electrostatic attraction. Moreover, UV-vis absorbance served to explain the interactions of PSNPs with four metals. FTIR and 2D-COS analyses confirmed the strong hydrogen bonding between the hydroxyl group of goethite and PSNPs with higher adsorption of PSNPs on goethite than magnetite. Additionally, ζpot measurements served to determine the point zero charge in the presence of different metals and iron oxides. Then, adsorption isotherms and related fitting parameters of Henry, Freundlich, and Langmuir models served to study adsorption kinetics.

J. Wang et al. [35] used sulfate-modified PSNPs to assess the influence of several factors on its aggregation; considering NOM present in aqueous media (SRHA and SRFA), TOC contents were characterized. Then, aliquots of PSNPs suspensions were mixed with electrolyte solution (CaCl_2_, Na_2_SO_4_, and NaCl) with and without NOM. Morphology of PSNPs was registered by TEM and ζpot. The sedimentation was followed by time-resolved optical absorbance at 269 nm. Fluorescence intensities were measured by excitation–emission matrix (EEM) fluorescence spectroscopy. XPS served the authors to determine the surface composition of PSNPs. The concentration of metal and anions was characterized by ICP-OES and IC, respectively. Aggregation and long-term stability were determined by TR-DLS.

Y. Zhang et al. [36] reported on the removal efficiency of MNPs during drinking water treatment. To achieve their results, the authors followed methods such as the CFS test, which was carried out with raw water samples from WTP, adding different fluorescent particles, using the Phipps and Bird programmable jar test. Then, to perform turbidity tests in triplicate, researchers added the coagulant (Al_2_(SO_4_)_3_ at 20 ppm). The sediments were cached and filtered using filter membranes (with pore sizes ranging from 0.2 to 0.025 μm). Additional CFS experiments were performed with coagulant aid poly-diallyldimethylammonium chloride (PolyDADMAC (20 ppm)) to test the increase in particle collision and thus enhance removal. To complete the filtration process, authors approached bench-scale filtration units to replicate the standard pilot-scale drinking water filtration system at the reference WTP. Moreover, the interactions of biofilms and microplastics was also tested to confirm the effect of biofilms on CFS and filtration processes. A standing microscope in tandem with a Nikon Ds Fi3 camera and suitable software was used for membrane image processing under green, red, or blue light for different fluorescent particles tested.

**Figure 4:**
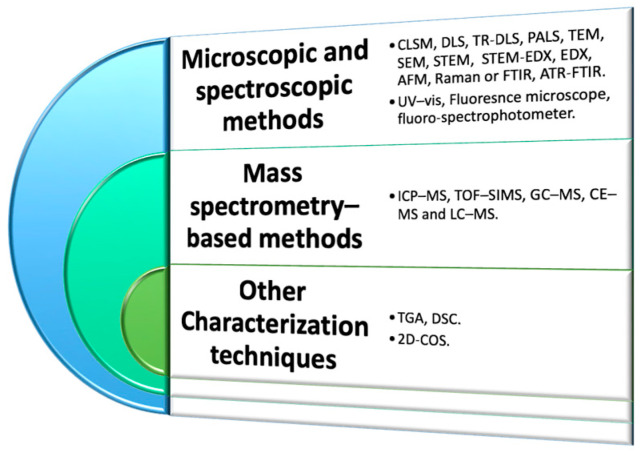
Different techniques used for MNPs characterization.

Tiwari et al. [37] synthesized Zn-Al LDH by precipitation method, then LDH were characterized by FESEM, TEM, and subsequently, ζpot and pH_PZC_ of NPDs + Zn-Al LDH using a DLS equipped with a pH titrator. Then, porosity and surface area measurements of the Zn-Al LDH were recorded with nitrogen as absorbate on Quantachrome Quadrasorb automatic volumetric instrument, then degassed under vacuum. The surface area was determined by a BET method. The crystalline structure of Zn-Al LDH was recorded by PXRD. For sorption studies, the researchers used a fluorescence spectrometer, while for the isotherm studies, NPDs concentration was minimized to avoid the self-aggregation of LDH. Figure 4 provides a summary of the main techniques used to characterize MNPs.

### 2.3. Current NPs’ Removal Efficiency

Advances in the efficiency of removing microplastics and nanoplastics (180 nm–125 μm) during drinking water treatment were reported [33]. These authors studied coagulation/flocculation combined with sedimentation (CFS) and granular filtration under usual running conditions at water treatment plants (WTPs). The interactions between microplastics and biofilms and the consequent influence on treatment efficiency were also studied. Mostly, CFS was not enough to remove MNPs. The effect of coagulant Al_2_(SO_4_)_3_ served to achieve a sedimentation rate of clean plastics lower than 2.0% for all different sizes of plastic particles. Even with adding coagulant aid (PolyDADMAC), the highest removal was only 13.6% for 45–53 μm of particles.

While granular filtration was much more effective at filtering out MNPs, from 86.9% to nearly complete removal (99.9% for particles larger than 100 μm); however, for a critical size (10–20 μm), a noteworthy lower removal (86.9%) was observed. Moreover, biofilm growth meaningfully augmented the removal efficiency of CFS treatment from 2.0% to 16.5%. 

Removal of nano-scale plastic debris from an aqueous environment by eco-friendly adsorption techniques was first reported by Tiwari et al. [37]. Nano-scale plastic debris (NPDs) incipient potential pollutants as they can be easily consumed by aquatic organisms and transport many contaminants into the environment [1]. Initially, the interaction between NPDs and synthesized Zn-Al layered double hydroxide (LDH) was confirmed by pH titration of Zn-Al LDH against NPDs at varying mass ratios (50:1 to 50:7) and FTIR analysis for both before and after 2 h of contact time. The removal efficiency was 100% at pH 4; at pH 9, it reached 37% due to increased competitive binding and destabilization of LDH under alkaline conditions. The process of sorption was spontaneous in different types of water studied. The study revealed that Zn-Al LDH could be a potential adsorbent to remove NPDs from freshwater systems. A summary of important information regarding the nature of NPs characterization methods and results in aqueous media with several chemistry and additives is shown in Table 1.

## 3. Discussion

The composition of plastics is highly variable, depending on their intended use. The density of NPs varies depending on their composition. Additional factors govern the stability of dispersions of nanoparticles in aqueous media, such as the core composition, surface chemistry, and solution conditions. 

The surface chemistry promotes the enhancement of PLNP aggregation when DOC was present and was associated with the bridges’ formation in the divalent cation solutions. Other research focused on monovalent co-ions were IO^−^, F^−^, Cl^−^, NO^−^, I^−^, and SCN^−^. For these co-ions, the ordering of the ccc (critical coagulation concentration) values was reversed when using the kosmotrope sodium and the chaotrope potassium or ammonium counterions comparatively. Their results suggested the competitive behavior of co-ions and counterions within the inner layer at the hydrophobic particle–aqueous solution interface. 

The authors focused on the role of surfactants on the stability of an NPs dispersion and reported that the particle size did not depend much on the amount and surfactant type (Tween 60, Tween 80, and a biosurfactant). It must be noted that PE particles remained stable in aqueous solutions for at least three months and did not aggregate in water containing sea salt at the average marine concentration (~35‰). The authors concluded that the aggregation of PS NPs with Fe (III) leads to that with Na(I) and Ca(II), and the subsequent effect of NOM depends on specific solution conditions (such as pH and ionic strength).

The surface modification and nature were studied using three monodisperse NPs, including unmodified NPs, carboxylated NPs, and amino-modified NPs, among one polydisperse NPs produced by laser ablation of polystyrene films, which were used as models to clarify the significance of morphology and surficial groups. Their results highlight that surface charge and surface modification notably impact the aggregation behaviors of NPs in aquatic systems. The Derjaguin–Landau–Verwey–Overbeek (DLVO) theory explains the aggregation phenomena of NPs with different functional groups and humic acid in the surrounding media. The PS NPs can interact with organic matter to form large organic particles, which may undergo further settling in response to specific salinity levels. Polystyrene-induced microgel formation involves the hydrophobic interactions between plastics and DOM. They showed that NPs alter the DOM–POM shunt to cause unexpected perturbations in aquatic ecosystems’ functionality. The aggregation kinetics of nano-PS clearly showed reaction- and diffusion-limited regimes in the presence of different metal cations. The CCC for different valence ions was consistent with the Schulze–Hardy rule. The decrease in the energy barrier and second energy minimum caused by metal cations contributed to nano-PS aggregation. The CCC of the nano-PS in the presence of NaCl at different pHs agreed with the DLVO theory. The aging-induced C=O groups’ formation on the surface of the nano-PS reduced the aggregation rates. The interaction amid the nano-PS particles in the presence of EPS (extracellular polymeric substances) was dominated by steric hindrance, electrostatic repulsion, and Van der Waals forces. These results can aid in understanding the aggregation behavior of nano-plastics in aquatic environments.

## 4. Conclusions

Given previously described research, we can conclude that the aggregation of NPs depends on the polymer nature of the NPs (resin type), surface chemistry (with or without functional groups), salinity and surrounding media conditions (pH, ionic strength), and presence of organic matter, both additives (acting as stabilizers or capping agents) or natural occurring matter (including, dissolved organic matter, dissolved organic carbon, and extracellular polymeric substances).

The behavior of nanoplastics showed that the PS/AOT/water system’s charging mechanism was adsorption-dominated, revealing the importance of the association between the activation of the particle surfaces and particle charging.

In addition, Mitrano et al. expect that their approach (synthesizing metal-doped nanoplastics) can be further developed to assess the fate, transport, mechanistic behavior, and biological effects at various systems on different scales. These results clarify the PSNP (nanoplastics’ (PSNPs) interactions with four typical minerals, comprising goethite, magnetite, kaolite, and montmorillonite aqueous phase, were studied.) with mineral surfaces and the potential outcome of PSNP on natural conditions in aquatic systems.

NOM was the most significant factor affecting the NPs aggregation for high ionic strength and NOM content water, probed through initial and long-term stability studies. PSNPs would remain highly suspended in all studied freshwaters, even in wastewaters, while they would aggregate quickly and sediment in seawater. The results confirmed that steric repulsions and electrostatic interactions dominated the aggregation of PSNPs. At low ionic strength, co-ions played a crucial role in providing charges on nanoplastics surfaces, while at high ionic strength dominated the electronic double-layer compression and charge neutralization by counterions.

## 5. Future Perspectives

The latest research on MNPS removal and directions to take are summarized in this section. In this sense, H. Zhao et al. [36] proposed a novel magnetic material, fly ash, as a biological raw material to be modified with iron ions to remove PSNPs from aqueous solutions. This new material showed as a suitable material for surface and pore attachment of PSNPs. They tested several pHs from weakly acidic to neutral, favoring the adsorption of NMPs. Thermodynamic, isothermal, and kinetic models used to analyze the possible adsorption mechanism lighted the complexation, π–π interaction, and electrostatic attraction, which are involved in the adsorption mechanism. It has been previously reported that predictive studies [38] based on the characteristics of MNPs and those of the contaminants themselves help address the transport of pollutants associated with these “Trojan horses”. Additionally, the NMA (new magenetic material) adsorbents showed excellent reusability for PSNPs entrapment [39]. Another magnetic material was proposed by X. Shi, X. Zhang, W. Gao, et al. [40] to remove microplastics from water using Fe_3_O_4_ nanoparticles via surface absorption (1.3 g.L^−1^, for 150 min). Magnetized MPs can be removed by aspirating the magnet. The authors tested with four types of MPs, such as PE, PP, PS, and PET, with sizes ranging from 200 to 900 μm, with the removal efficiency higher than 62% for all MPs tested. The removal rate varied between the polymer type and particle size of MPs and was positively related to the amount of Nano-Fe absorbed at MPs surface. 

A.G. Mukherjee et al. [41] reviewed the importance of the interaction of MNPs with other pollutants present in water sources. They highlighted that the MPs are produced in small sizes for specific applications. Proposed and reported membrane technologies are very effective for MPs contaminants removal. Moreover, the hybrid membrane bioreactors show a pretty lower layer fouling. Finally, the authors reviewed and approached that the biofouling may be merged with dynamic membrane technology for MPs removal. A different approach based on CuNi carbon material has been proposed by G. Zhou et al. [42] to remove PSNPs from water systems. The authors underlined the solo of adsorption. CuNi@C carbon material was prepared by hydrothermal method to remove polystyrene (PS) nanoplastics from water. A slight change in the concentration of CuNi@C from 0.1 to 0.3 g/L promotes an increase in the removal efficiency of NPs from 32.72 to 99.18%. SEM images and FTIR characterization confirmed the adsorption of PSNPs on CuNi@C. The results of adsorption kinetic models and isotherms equations proved that monolayer coverage and physical adsorption were the predominant mechanisms of the PSNPs’ adsorption on CuNi@C.

Future lines of research must add all the necessary stakeholders, including researchers from the R + D + i sector, whether chemistry, chemical engineering, environmental engineering, biochemistry, etc., as well as the work of waste managers, local, regional, national, or international governments, coupled with the work of each individual to minimize the use of plastic materials, to advance hand in hand by the sum of the efforts of all the actors involved in the use and management plastic waste. 

## Figures and Tables

**Table 1:** Summary of important information regarding the nature of NPs characterization methods and results in aqueous media with several chemistry and additives.

NPs’ Nature and Size	NPs’ Modification	Dispersion Medium	Characterization Methods and Other Experiments	Size of Aggregates/Method Efficiency	Ref.
polystyrene latex nanoparticles (PLNPs)	− unmodified (uPLNPs), − amine-modified (aPLNPs), and − carboxyl-modified (cPLNPs)	− DOC increased the surface charge and exhibited negligible effects on the size distribution of the PLNPs in aqueous suspensions. − divalent cations (Ca^2+^ and Mg^2+^). − The coexistence of DOC and the divalent cations enhanced the extent of aggregation of the PLNPs in the water columns.	DLS, TEM, UV-vis.	− *D_H_* of the PLNPs decreased in the order of uPLNPs > aPLNPs > cPLNPs. − The enhancement of PLNP aggregation when DOC was present was associated with forming a bridge in the divalent cation solutions.	[16]
PS, 468 nm with a 0.08 PDI	-	Ions and co-ions. The ranking of CCC was IO_3_^−^ ≤F^−^ < Cl^−^ < NO_3_^−^ < I^−^ < SCN^−^.	DLS, SEM, UV-vis.	Competitive behavior of the implied co-ions and counterions within the inner layer at the hydrophobic particle–aqueous solution interface.	[20]
Polyethylene particles with radii between 200 and 800 nm	-	The surfactants used to synthesize nanoplastics were Tween 60, Tween 80, and a biosurfactant. Emulsification of PE in toluene solutions in water.	CLSM, TGA, DSC, Bioavailability of PE particles in the presence of *Daphnia magna.*	-	[21]
Yellow-green, fluorescent Polystyrene nanoparticles with a diameter of 0.1 μm.	-	− NaCl solutions at 1-, 10-, 50-, and 100-mM ionic strengths. − NOM: SRHA and − SRFA in CaCl_2_ solutions with different ionic strengths − PS NPs for 50 mg L^−1^ PS NPs in FeCl_3_ solutions at ionic strengths of 0.01, 0.1, and 1 mM. − Fluorescence microscope images and Cryo-SEM images.	DLS, fluorescence microscope, Cryo-SEM.	− Inorganic salts (NaCl and CaCl_2_) and NOM (SRHA and SRFA) had negligible effects on aggregating PS NPs in solutions at environmentally relevant conditions. − The sizes of PS NPs increased significantly over time in FeCl_3_ solutions (reaching ~350 nm) with ionic strengths of 0.1 and 1 mM. − The size of PS NPs reached ~1200 nm after 10 min in 1 mM FeCl_3_ solution in the presence of either SRHA or SRFA.	[22]
Polystyrene beads (50 nm).	− no surface functionalization (plain) − carboxylic − amine functionalization	− ultrapure water (UW; pH 6.6 ± 0.2); − artificial seawater (ASW; pH 8.1 ± 0.1; 30 practical salinity unit (PSU); NaCl 450 mM, KCl 10 mM, CaCl_2_ 9 mM, MgCl_2_ 30 mM and MgSO_4_ 16 mM); − 2 mm filtered natural seawater from the Bay of Brest sampled in January 2018 (FSW; pH 8.2 ± 0.1; PSU 32). − Salinity gradient (from 0 to 653 mM). − range 1–30 μg.L^−1^ and 1–10 μg.L^−1^ of organic matter in artificial seawater and ultrapure water.	DLS.	− The negatively charged carboxylic and plain nanobeads’ stability was lost under an increasing ionic strength, resulting in homo-aggregation (up to 10 μm). − The increase in organic matter content had a negligible effect on these two nanobeads. − Particles stayed at a nanometric scale (PS-COOH; 63.4 ± 3.43 nm; PS-Plain: 56.0 ± 0.2 nm; PDI < 0.2) only in UW and formed microscale aggregates in ASW (PS-COOH: 1835.0 ± 240.0 nm; PS-Plain: 2106.7 ± 75.4 nm) and FSW (PS- COOH: 4530.3 ± 528.0 nm; PS-Plain: 4810.3 ± 370.2 nm).	[23]
92.8 ± 6.9 nm, 97.3 ± 7.8 nm, 109.2 ± 8.5 nm, 60.2 ± 26.3 nm - data obtained by SEM-.	PS-Bare, PS-COOH PS-NH_2_ PS-Laser	mono- and divalent cations with and without SRNOM.	DLS, SEM, aggregation kinetics.	Attachment efficiency.	[24]
− polystyrene NPs The nominal size of PS is 100 nm (TEM), while the hydrodynamic diameter of the PS NPs measured by DLS is approximately 220 nm. − fluorescent PS microspheres with a diameter of 100 nm	different functional groups and charges (PS, PS-COOH, n-PS-NH_2_, p-PS-NH_2_)	Humic acid (HA) and salinity.	TOC analyzer, TEM, DLS, for ζpot used PALS, elemental analyzer; fluorescence quenching of PS was carried by fluoro-spectrophotometer, a 48 h acute toxicity test on *Daphnia magna* was performed following the OECD guideline 202 (OECD, 2004).	The observations indicated that the stability, transport, and toxicity of NPs in aquatic systems were highly dependent on environmental factors (effect of HA content and salinity).	[25]
25 nm polystyrene nanoparticles	-	Lake and river water can promote POM (microgel) formation and accelerate the DOM–POM transition. Authors adjusted various salinities of water samples to simulate plastic transport scenarios in waters flowing from rivers to seas. The sea salt (NaCl, MgSO_4_, MgCl_2_, CaCl_2_, KCl, and NaHCO_3_) To further examine the hydrophobic interactions and possible intermolecular cross-linking between DOM and polystyrene, EDTA was added to DOM samples (with/without polystyrene).	DLS, no subsequent micron-scale aggregates present (135.8 ± 79.66 nm).	Polystyrene-induced microgel formation appears to involve the hydrophobic interactions between plastics and DOM. The results indicate that polystyrene nanoparticles can interact with organic matter to form large organic particles. DOM polymers from both sources exhibited a similar assembly curve, reaching microgels of roughly 4–6 μm within 90–120 h.	[26]
Polystyrene nanoplastics (nano-PS). The nominal bead size of PS was 100 nm.	Nano-PS was artificially aged in an aging chamber equipped with two symmetrical UV lamps (UVC-254 nm, 15 w)	− Evaluate the effects of the various metal cations (K^+^, Na^+^, Ba^2+^, Ca^2+^, Mg^2+^, Fe^3+^, Al^3+^) on the aggregation of nano-PS. − Aggregation characteristics of nanoplastics under different pH conditions (3.5, 5.5, 7.5, and 9.5). − Assess the influence of EPS (excreted from *Chlorella pyrenoidosa*, a common freshwater algae species) on nano-PS aggregation.	After the aging process, DLS, SEM, and ATR-FTIR changes were evaluated using X-ray photoelectron spectroscopy, ATR-FTIR spectra, and thermogravimetric-differential scanning calorimetry. Time-resolved DLS followed aggregation kinetics.	The hydrodynamic size of nano-PS was 113.8 nm with a range of 58.8–190.0 nm, close to the average diameter observed from the SEM images (104.9 ± 0.76 nm). At the end of the testing period, the hydrodynamic sizes of the nano-PS (more than 600, 1100, and 1200 nm in 400 mM, 500 mM, and 600 mM NaCl, respectively) in solutions without EPS were significantly larger than the hydrodynamic sizes of the nano-PS (less than 400, 800, and 1000 nm in 400 mM, 500 mM, and 600 mM NaCl, respectively) in solutions with EPS, which could be related to steric effects.	[27]
Poly(vinyl acetate) (PVAc) latex particles. The size was found to be lower than 200 nm.	NPs were coated with natural polyelectrolytes (alginate and chitosan) via layer-by-layer deposition.	The SDS was used as an emulsifier during the miniemulsion polymerization process.	DLS and TEM highlighted a spherical shape and a size lower than 200 nm. UV–vis.	The implemented strategy offers the advantage of ease and efficiency of nanocarrier elaboration via a scalable process, and the versatility of their preparation makes them adaptable to a range of applications. The process’s efficiency prevents any residual monomer from remaining in the colloids, avoiding toxicity issues.	[28]
Polystyrene (PS) particles. Three different sizes: 141, 311, and 535 nm.		NPs were dispersed in aqueous solutions of the surfactant sodium di-2-Ethylhexyl sulfosuccinate (AOT).	DLS; using PALS.	At different AOT concentrations (0.1–10 mM), the size of the PSNPs was 535 nm.	[29]
The particles synthesized were polyacrylonitrile (PAN) core material, which contained the metal tracer, followed by the addition of a cross-linked polystyrene (PS) shell.	The reasoning for this multi-step process was to impart a few key benefits over a single batch emulsion, including acrylonitrile (AN), which is capable of chemically complexing the Pd in the water phase; the core/shell structure would ensure there was minimal leaching of metal from the particle, and the shell could be changed independently of the core.	Batch studies were performed to simulate a municipal WWTP’s activated sludge process using a continuously running pilot-scale system on-site using actual municipal wastewater.	− Imaging (SEM, STEM-EDX), − sizing (DLS, electron microscopy), − metal (Pd) incorporation (ICP-MS).	Three independent objectives were: (1) evaluating the evenness of particle distribution in well-mixed activated sludge (and thereby optimizing sampling protocol for nanoplastics); (2) determining the affinity for heteroaggregation of nanoplastics with the suspended solids in the sludge (i.e., surface affinity and how quickly nanoplastics adhere to sludge flocs); and (3) assessing total nanoplastic retention in WWTP.	[33]
Polystyrene nanoplastic (PSNP)	Incubation of PSNP with other three metal oxides with different surface charges, MnO_2_, Al2O_3_, and SiO_2_	Four typical minerals, including goethite, magnetite, kaolinite, and montmorillonite, in the aqueous phase were investigated.	TEM, SEM, FTIR, 2D-COS; UV-Vis Absorbance. The ζpot of the three particles as a function of the pH suspension and the PZCs (point of zero charges).	− Stability of PSNP colloidal suspension was reduced by goethite and magnetite. − Electrostatic attraction drove PSNP to attach with the positively charged minerals. − The adsorption capacity of PSNP on goethite was higher than on magnetite. − Hydrogen bonding contributed to the adsorption of PSNP on goethite.	[34]
Polystyrene (PS) NPs	The PS NPs *D_H_* remained relatively constant (90–100 nm) from pH 2.3 to 11.1.	Different hydrochemical conditions such as pH, salt type (NaCl, CaCl_2_, Na2SO_4_), ionic strength (IS), and natural organic matter (NOM: SRHA, SRFA) were studied.	TEM, DLS, and TR-DLS, UV-Vis spectroscopy. The PS NPs’ surface composition was determined by X-ray photoelectron spectroscopy. The TOC contents of NOM and natural waters were quantified using the TOC analyzer.	The isoelectric point (pHzpc) of PS NP was <2.3. The ζ-potential of PS NPs was for pH 2.5 to 5.4 (from −15.4 ± 165 0.9 mV to −38.6 ± 0.4 mV) and for pH 5.4 to 11.1 (from −38.6 ± 0.4 mV to −49.6 ± 1.2 mV)	[35]
Microplastics and nanoplastics (80 nm, 1.2 μm, 10–20 μm, 45–53 μm, and 106–125 μm)). One was polyethylene (PE) (10–20 μm, 45–53 μm, and 106–125 μm), and the other was polystyrene (PS) (180 nm and 1.2 μm).	-	Coagulant Aid PolyDADMAC, biofilm.	CFS with Coagulant Aid PolyDADMAC. Filtration. Interactions of biofilms and microplastics.	The authors characterized the removal efficiency of micro- and nanoplastics (180 nm–125 μm) during drinking water treatment. Future research should focus on removing small-sized plastics (10–20 μm) in water treatment processes because these particles are more difficult to remove yet could cause more significant health concerns if ingested.	[36]
Polystyrene (PS) nanoparticles (d = diameter of the particles is 55 nm and hydrophilic nature as coated with an anionic surfactant to make them uniformly disperse in water).	The synthesized Zn-Al layered double hydroxide (LDH) was confirmed by pH titration of Zn-Al LDH against NPDs at varying mass ratios (50:1 to 50:7).	ZnCl_2_, Al(NO_3_)_3_.9H_2_O, NaOH, NaNO_3_, Na_2_SO_4_, NaHPO_4_, NaHCO_3_, and NaCl. Synthesis of Zn-Al LDH	FTIR analysis for both before and after 2 h of contact time.	Sips isotherm observed fast removal in deionized and synthetic freshwater with a maximum sorption capacity (Qmax) of 164.49 mg/g and 162.62 mg/g, respectively. Removal was least in synthetic hard water, with a Q_max_ value of 53 mg/g. For 2 mM concentrations of SO_4_^2−^, and PO_4_^3−^, the adsorption capacity significantly decreased to 2%. The removal efficiency was found to be 100% at pH 4.	[37]

## Data Availability

Not applicable.

## Abbreviations

2D-COS	Two-dimensional correlation spectroscopy
3D-EEM	Three-dimensional fluorescence excitation–emission matrix
AOT	Sodium di-2-Ethylhexyl sulfosuccinate
aPLNPs	Amine-modified polystyrene latex NPs
ATR-FTIR	Attenuated total reflectance Fourier transform infrared spectroscopy
BET	Brunauer–Emmett–Teller
BPA	Bisphenol A
CCC	The critical coagulation concentration
CE–MS	Capillary electrophoresis–mass spectrometry
CFS	Coagulation/flocculation combined with sedimentation
CLSM	Confocal laser scanning microscopy
cPLNPs	Carboxyl-modified polystyrene latex NPs
*D_H_*	Hydrodynamic diamter
DLS	Dynamic Light Scattering
DLVO	Derjaguin–Landau–Verwey–Overbeek
DOC	Dissolved organic carbon
DSC	Differential scanning calorimetry
EDTA	Ethylenediaminetetraacetate
EDX	Energy dispersive X-ray
EPS	Extracellular polymeric substances
FTIR	Fourier Transform infrared spectroscopy
GC–MS	Gas chromatography–mass spectrometry
HA	Humic acid
HCH	Hexachlorocyclohexane
ICP-MS	Inductively coupled plasma–mass spectrometry
ICP–MS	Inductively coupled plasma–mass spectrometry ()
LC–MS	Liquid chromatography–mass spectrometry
LDH	Layered double hydroxide
MNPs	Micro- and nano-plastics
MPs	Microplastics
n-PS–NH_2_	Negatively charged amino-modified Polystyrene nanoplastics
NOM	Natural organic matter
NPDs	Nano-scale plastic debris
NPs	Nanoplastics
p-PS–NH_2_	Positively charged amino-modified Polystyrene nanoplastics
PAHs	Polyaromatic hydrocarbons
PALS	Phase angle light scattering
PBTs	Persistent bioaccumulative and toxic compounds
PCBs	Polychlorinated biphenyls
PE	Polyethylene
PET	Polyethylene terephthalate
pHpzc	Isoelectric point or point zero charge
PLNPs	Polystyrene latex NPs
PolyDADMAC	Poly-Diallyldimethylammonium Chloride
POM	Particulate organic matter
POPs	Persistent Organic Pollutants
PP	Polypropylene
PS	Polystyrene
PS-COOH	Carboxyl-modified polystyrene
PU	Polyurethane
PVAc	Poly(vinyl acetate)
PVC	Polyvinyl chloride
PXRD	Powder X-ray diffraction
PZCs	Point of zero charges
SDS	Sodium dodecyl sulfate
SEM	Scanning electron microscope
SRFA	Suwannee River fulvic acid
SRHA	Suwannee River humic acid
SRNOM	Suwannee River natural organic matter
STEM	Scanning transmission electron microscope
STEM-EDX	Scanning transmission electron microscope -Energy dispersive X-ray
TEM	Transmission Electron Microscopy
TGA	Thermogravimetric analysis
TGDSC	Thermogravimetric-Differential scanning calorimetry
TOC analyzer	Total organic carbon analyzers
TOF–SIMS	Time-of-flight secondary ion mass spectrometry
uPLNPs	Unmodified polystyrene latex NPs
UV-Vis	Ultraviolet-Visible spectrophotometer
WTP	Water treatment plant
WWTP	Wastewater treatment plant
XEDS	X-ray energy-dispersive spectrometer
XPS	X-ray photoelectron spectroscopy
ζpot	Zeta potential
